# Virtual reality hypnosis diminishes experimental cold pain and alters autonomic responses

**DOI:** 10.3389/fpain.2023.1237090

**Published:** 2023-11-02

**Authors:** Claire Terzulli, Chloé Chauvin, Cédric Champagnol Di-Liberti, Sylvain Faisan, Laurent Goffin, Coralie Gianesini, Denis Graff, André Dufour, Edouard Laroche, Eric Salvat, Pierrick Poisbeau

**Affiliations:** ^1^ Centre National de la Recherche Scientifique, University of Strasbourg, Institut des Neurosciences Cellulaires et Intégratives, Strasbourg, France; ^2^ HypnoVR, Strasbourg, France; ^3^ Anesthesiology and Intensive Care, University Hospital of Strasbourg, Strasbourg, France; ^4^ ICube Laboratory, University of Strasbourg, Strasbourg, France; ^5^ Anesthesiology Department, Clinique Rhéna, Strasbourg, France; ^6^ Centre National de la Recherche Scientifique, University of Strasbourg, Laboratoire de Neurosciences Cognitives et Adaptatives, Strasbourg, France; ^7^ Chronic Pain Center, Hopitaux Universitaire de Strasbourg, Strasbourg, France

**Keywords:** virtual reality, hypnosis, pain, analgesia, autonomic changes, thermal pain

## Abstract

Immersive virtual reality (VR) is a promising tool to reduce pain in clinical setting. Digital scripts displayed by VR disposals can be enriched by several analgesic interventions, which are widely used to reduce pain. One of these techniques is hypnosis induced through the VR script (VRH) which is facilitated by immersive environment and particularly efficient even for low hypnotizable patients. The aim of this study is to assess the efficacy of a VRH script on experimentally induced cold pain perception (intensity and unpleasantness) and physiological expression. 41 healthy volunteers had been recruited in this within-subjects study. They received 9 stimulations of 20 s (3 non-nociceptive cold; 3 low nociceptive cold and 3 highly nociceptive cold) during a VRH session of 20 min (VRH condition) or without VRH (noVRH condition). Physiological monitoring during the cold pain stimulation protocol consisted of recording heart rate, heart rate variability and respiratory frequency. Maximum cold pain intensity perception, measured through the visual analog scale (VAS) on 10, was of 3.66 ± 1.84 (VAS score/10) in noVRH condition and 2.46 ± 1.54 in VRH (Wilcoxon, *p* < 0.0001). Considering pain unpleasantness perception, 3.68 ± 2.06 in noVRH and 2.21 ± 1.63 in VRH (Wilcoxon, *p* < 0.0001). Hypnotizability negatively correlated with the decrease in VAS intensity from noVRH to VRH (Spearman *r* = −0.45; *p* = 0.0038). In our sample, we found that 31/41 volunteers (75.6%) displayed a reduction of more than 10% of their VAS pain intensity and unpleasantness scores. Trait anxiety was the best predictor of the VRH responders, as well as heart rate variability. In addition, respiratory rate was diminished under VRH in every subgroup. VRH is an effective tool to reduced pain intensity and unpleasantness in a vast majority of healthy subjects. We further indicate in this study that heart rate variability parameter RMSSD (root mean square of successive differences) is a good predictor of this effect, as well as anxiety as a personality trait (but not state anxiety). Further studies are expected to determine more precisely to whom it will be the most useful to offer tailored, non-pharmacological pain management solutions to patients.

## Introduction

Head-mounted virtual reality (VR) devices that use a computer-generated three-dimensional world in which one is immersed and in which stimuli can be multisensory (e.g., visual, auditory, and/or haptic) offer a real opportunity for alleviating acute/chronic pain in adults and children (1). For more than two decades, many VR devices have been evaluated thanks to the pioneering work of Hoffman and Patterson (2, 3). Their initial studies examined procedure-related pain in burn patients while using a VR device that allowed active participation in a game (Snow World). With this device, a 30%–50% reduction in procedural pain was observed for several pain outcomes such as pain intensity, unpleasantness or time thinking about pain (2), and this effect correlated with a significant reduction in hemodynamic responses in at least five regions of the pain matrix: the anterior cingulate cortex, primary and secondary somatosensory cortex, insula, and thalamus (4, 5). Regardless of the content of the disposal, VR has demonstrated some efficacy as a complementary technique to reduce acute pain and anxiety associated with various medical procedures (6–10), as well as in the treatment of chronic pain (11). Although promising, VR is still an emerging technology, its efficacy needs to be consolidated through further scientific studies, especially for acute and chronic pain management. Moreover, it remains to optimize analgesic intervention of VR and potentially better understand the underlying brain mechanisms that lead to pain relief. Notably, there is currently a lack of consistency across studies regarding VR outcomes in pain alleviation (7, 8). Most of these are related to the heterogeneity of methodological approaches, the variety of digital scripts embedded in VR, the choice of control groups, small sample size, or the selection of subjects/patients for pain assessment (12).

While VR pain management applications often rely on distraction techniques (either passively with movies or actively with games; the difference lies in the degree of interactivity) as they mobilize multiple inhibitory pain controls (1), they can also be used with other analgesic approaches such as cognitive behavioral therapy (11), meditation (13), hypnosis (14–16), or biofeedback (17). In these options, patients become actively involved in managing their own pain by shifting their focus or improving their skills (18).

Combining hypnotic suggestions to VR disposal has raised a lot of interest. Hypnosis can be defined as a modified state of consciousness with increased focused attention and reduced peripheral awareness, characterized by an increased ability to respond to suggestions (19). Although the clinical use of hypnosis is steadily increasing, there are interindividual differences in hypnotizability (i.e., a person's ability to experience suggested changes in physiology, sensations, emotions, thoughts, or behavior during hypnosis) (19). Hypnotizability scores, which can be assessed with specific scales (such as the Stanford scale), are normally distributed in the population (20–23). Thus, hypnosis efficacy varies in the general population. To bypass this issue, one solution can be to combine hypnosis with VR (referred in this manuscript to as VRH, for Virtual Reality Hypnosis), i.e., using a virtual environment to help patients access this particular state. VRH appears to be more beneficial for individuals with low hypnotic ability than for individuals with high hypnotic ability, whereas VR alone is not as efficient as hypnosis for individuals with high hypnotic ability (15, 24). However, one study showed VRH to be more effective than VR alone in relieving pain but not hypnosis alone (14), whereas Rousseaux and colleagues found no benefit of VR, hypnosis, nor VRH for pain and anxiety reduction in cardiac surgery patients compared with standard care (25).

To further support the interest of using VRH as an analgesic strategy, monitoring physiological functions is often used to support the subjective benefits of VRH on well-being, pain relief and anxiolysis. Indeed, VR disposals are likely to affect homeostatic parameters regulated by the autonomic nervous system (ANS). Heart rate variability (HRV), measured as changes in standard deviation from normal to normal (SDNN) and root mean square of successive differences (RMSSD), have been shown to be modulated by VR (26–29). Using a frequency domain analysis of the electroencephalogram (ECG), VR-induced changes of the low frequency (LF) and low to high frequency ratio (LF/HF), thought to reflect the regulatory balance between the parasympathetic and sympathetic tone (low ratio: predominance of parasympathetic, high ratio: dominance of sympathetic), were also seen (28, 30).

In this scientific setting, we examined the efficacy of VRH on experimentally induced cold pain, looking closely at how VRH affects pain perception. Effects were correlated with changes in cardiac and respiratory parameters in an attempt to identify predictors of VRH efficacy.

## Methods

### Participants

After ethic committee approval (CPP Ile-De-France III, approval date 04/02/2020; ANSM French Ministry of Health, information date 23/05/2019) and signed written consent, 41 participants were included, 19 females and 22 males. Healthy volunteers were older than 18 years old (see demographic data, Table 1). Exclusion criteria were unbalanced epilepsy, diseases that change pain perception (e.g., chronic pain conditions, diabetes), psychotic disorders, depression, hearing and/or visual impairments preventing the use of VRH, participating in another clinical study, in guardianship or unable to provide informed consent or refused to participate. Female subjects could not participate if they were pregnant or breastfeeding.

**Table 1:** Demographic data.

	Total (*n* = 41)	Female (*n* = 19)	Males (*n* = 22)	Statistics male vs. female
Age (years; mean ± SD)	41.3 ± 13.4	39.8 ± 12.4	42.6 ± 14.5	ns, *p* = 0.51
Education (years postbac; mean ± SD)	4.8 ± 2.1	4.4 ± 1.9	5.2 ± 2.2	ns, *p* = 0.21
STAI-Trait (score/80; mean ± SD)	36.8 ± 8.6	37.4 ± 7.9	36.3 ± 9.3	ns, *p* = 0.58
STAI-State (score/80; mean ± SD)	28.2 ± 6.6	29.7 ± 6.9	26.8 ± 6.1	ns, *p* = 0.15
Stanford (score/12; mean ± SD)	5.9 ± 3.0	6. 9 ± 2.9	5.2 ± 2.9	*, *p* = 0.048

STAI, state-trait anxiety inventory. Mann-Whitney statistical test between male and female subjects: ns, non-significant; *difference at *p* < 0.05.

### Primary and secondary outcomes

The primary endpoint of the study was to evaluate the effect of VRH on perceived cold pain intensity and unpleasantness, evaluated by Visual Analog Scale (VAS/10). Secondary endpoints included the analysis VRH-associated changes in several physiological parameters during the cold stimulation protocol (see paragraph below, data collection).

### Study design and parameters recorded

**Figure 1:**
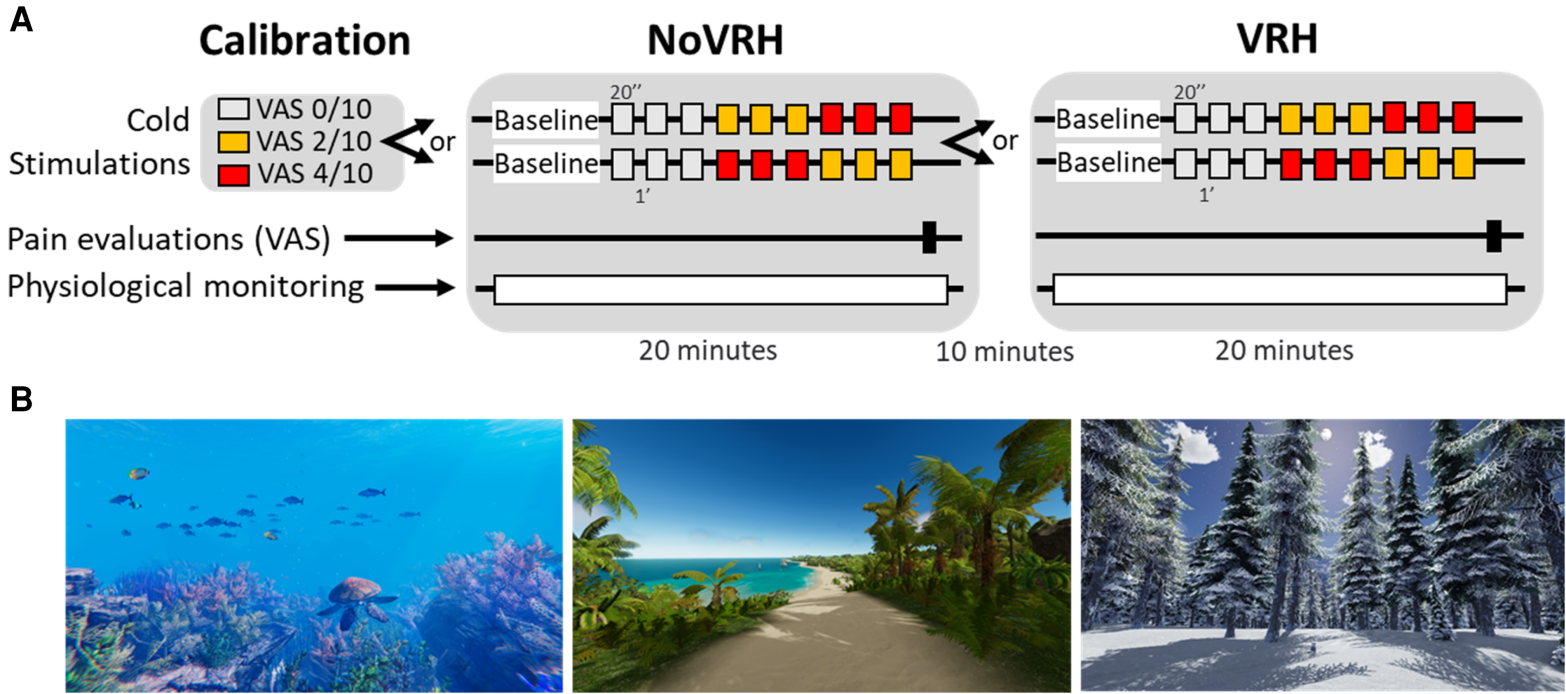
Experimental design of the study (**A**) and representative examples of some VRH environments (**B**). Subjects first determined the cold stimulation corresponding for them to a VAS intensity of pain of 0/10 (no pain), 2/10 (moderate pain) and 4/10 (high pain). This helped to adjust the corresponding cold nociceptive stimulation for each subject. Then, subjects entered a 20 min recording session, without any VRH disposal, where VAS pain intensities were asked after three consecutive cold stimulations corresponding to 0/10, 2/10 and 4/10. All stimulations lasted 20 s. Note that stimulations at 2/10 and 4/10 were randomly placed in the stimulation sequence. The second recording session corresponded to the exact same protocol but in the presence of the VRH disposal, which was installed during a five-minute interval. Physiological monitoring consisted of electrocardiogram recordings, respiration rates and electrodermal activity.

This was a prospective within-subjects study where each participant was his/her own control. General design of the study is shown in Figure 1A. Cold stimulations (20 s each, 1 min interval) of three different intensities, producing no pain on the VAS or pain intensities at 2/10 and at 4/10, were pre-defined during a calibration period (Figure 1A). During a first session of 20 min (noVRH), subjects were then submitted to a series of three stimulations at three different intensities (no pain, 2/10, 4/10). Note that the stimulation at 2/10 and 4/10 application timing was counterbalanced between participants (Figure 1A). At the end of the session, subjects were asked to evaluate their pain perception (intensity and unpleasantness) of the most painful stimulation. Physiological parameters monitored in this study were heart rate and its variability and breathing rate. During the second session, the same procedure was followed but subjects were equipped with the VRH disposal. It took 10 min maximum for the subject to be equipped. It is important to note here that sessions 1 and 2 were not randomized in this study as we previously demonstrated that randomization, with VRH or not, had no effect on pain measures or physiological parameters (29). For example, sequence order revealed no significant differences in VRH-induced changes for painful heat (noVRH-VRH: 0.86 ± 0.25, *n* = 28; VRH-noVRH: 0.63 ± 0.19; Student's *t*-test, *t* = 0.747; *df* = 56; *p* = 0.458).

### Virtual reality hypnosis

The VR headset used in this study was an Oculus Rift S (resolution: 1280 × 1440 pixels per eye; field of view: 115°; refresh rate: 80 Hz) coupled to a laptop computer (Asus GL502VS managed by an Intel®Core™ i7-6700HQ processor at 2.6 GHz; RAM: 16GB; graphics card: Nvidia GeForce GTX 1070; Windows 10 64-bit). The Oculus Rift S headset delivered sound.

The virtual reality application (referred to as VRH) used was HypnoVR® (www.hypnovr.io, Lampertheim, France). VRH is an application coupling three-dimensional computer-generated, immersive visual sceneries (either walk on the beach, scuba-diving, walk through a snowy mountain/forest, or a journey through space; see some representative screenshots Figure 1B) with a standardized pre-recorded 20 min hypnotic script tailored to induce analgesia and a musical background. The hypnotic script was the same for all visual environments. Participants were given the opportunity to choose their preferred visual scenery, male or female voice for hypnotic suggestions and musical background among three melodies. Hypnotic script consisted of an “induction period” (first three minutes) to focus participant's attention. In particular, they were asked to breathe at the same speed than a blue ball moving at a frequency 6 oscillations/minute. This oscillation frequency is known to have the greatest impact on heart rate variability parameters (i.e., cardiac coherence) (31). This induction period was followed by comfort and pain relief suggestions (including changing sensations from pain to something else, reduction in pain, increases in comfort, changes in focus attention away from pain and increased ability to ignore pain). The session ended with 2 min return phase to the normal conscious state.

### Thermal stimuli and experimental protocol

Cold stimuli were applied with a QST stimulator (TCS II, QST.Lab, Strasbourg, France, www.qst-lab.eu). Thermode was placed on the anterior part of the left wrist. Cooling surface was 10 cm² and decrease/increase speed of temperature was set at 15°C/s. Minimum temperature reachable with this device was 0°C. As mentioned above (see also Figure 1), the same stimulation protocol was applied during the noVRH and VRH sessions. Subjects did not know which temperature was chosen to obtain a VAS score of 2/10 and 4/10.

### Physiological data

The electrocardiogram (ECG) was acquired with BIOPAC ECG100C (BIOPAC System Inc, 42 Aero Camino, Goleta, CA 93117, USA). Two electrodes were placed on the participant's upper chest (one on each side) while the third one was placed on the ribs (left side). The breathing rate (cycles/min) was measured by means of a BIOPAC thermistor TSD202A (BIOPAC System Inc, 42 Aero Camino, Goleta, CA 93117, USA) which measured the temperature difference between inhaled and exhaled air; the thermistor was placed under the participant's nostrils and fixed with an adhesive plaster. Data were analyzed with Clampfit (Molecular Devices, LLC. 3860 N First Street, San Jose, CA 95134, United States) and script programed in Python 3.6. The baseline values were considered as the average of the first 5 min where no stimulation was applied. Mean values of each sequence of three stimulations were then averaged (0/10; 2/10; 4/10). Five minutes recording periods were needed to obtain a reliable low frequency extraction for heart rate frequency analysis. Therefore, the 5 min baseline period was compared to the period where cold stimulations were applied, including interval periods (of 1 min) between the stimulations.

### Data collection

Data were prospectively collected including demographic characteristics as age, sex, education level, number of previous hypnosis sessions and why (if any), motion sickness, hypnotizability score (French adaptation form A of the Stanford Hypnotic Susceptibility Scale (20–22), the anxiety trait was assessed on the day of inclusion using a self-administered questionnaire, the State-Trait Anxiety Inventory (STAI). On the day of the experiment, state anxiety was assessed with STAI-Y, current medication (if any) and adverse events. All the identifying information was removed from the database in accordance with regulations prescribed by the French data protection authority *Commission Nationale de l'Informatique et des Libertés (CNIL n° 2213128)*.

### Statistical analysis

Results are expressed as mean ± SD. The statistical analyses include a descriptive section and an analytical section. All the statistical analyses were made with Prism software Graphpad V6.0. The significance level was set at alpha = 0.05 for all analysis. Normality of the distributions was tested each time using the Shapiro–Wilk normality test. Differences between two groups were analyzed using the Student's *t*-test when appropriate when data were linearly distributed. Otherwise non parametric Mann-Whitney test was used to compare two populations. Two-way ANOVA (and repeated measures) were followed, if significant, by Sidak or Tukey multiple comparisons. Correlation between hypnotizability and pain score was assessed with Spearman's R correlation test.

## Results

### Population

On the 41 healthy volunteers included, 19 were females and 22 males. Mean age was 41.28 ± 13.43 years old with a range from 21 to 66 years old. Average education was 4.8 ± 2.09 years post-bac. Mean anxiety (personality trait) was of 36.85 ± 8.61 out of 80 whereas mean anxiety (current state the day of the experiment) was of 28.17 ± 6.58. Average hypnotizability score was 5.97 ± 3.01 out of 12, which is coherent with data in the general population (20–22). Of 41 persons, 9 (4 males, 5 females) have experienced at least one hypnosis session in their lifetime mostly because of anxiety issues (3/9). Other reasons were pain (1/9), sleeping issues (1/9), obsessional compulsive disorders (1/9) and 3/9 for unknown reasons. The only difference between male and female statistics was found for the hypnotizability score (5.19 ± 2.89 for male vs. 6.89 ± 2.97 for female; Mann-Whitney test, *U* = 119.5, *p* = 0.048). For detailed comparison, see Table 1.

### Calibration of the cold pain stimulation

During the calibration period preceding the VRH session, each subject was asked to score different cold intensities stimulations. This calibration allowed to define the stimulation intensities for each subject at three level: VAS value of 0/10 (no pain), pain at 2/10 and at 4/10. With the skin thermode used in this study, mean temperatures were of 24.95 ± 0.86°C (VAS 0/10), 10.80 ± 5.19°C (VAS 2/10) and 5.59 ± 5.23°C (VAS 4/10). We did find a statistical differences between males and females for cold threshold [two way ANOVA, sex x temperature, F_(2,117)_ = 5.189, *p* = 0,0069] for VAS at 2/10 (Tukey posthoc test, *p* = 0.0367 and 4/10 (Tukey posthoc test, *p* = 0.0009). Women had mean threshold for cold higher than males likely indicating a higher thermonociceptive sensitivity.

### Cold pain perception

**Figure 2:**
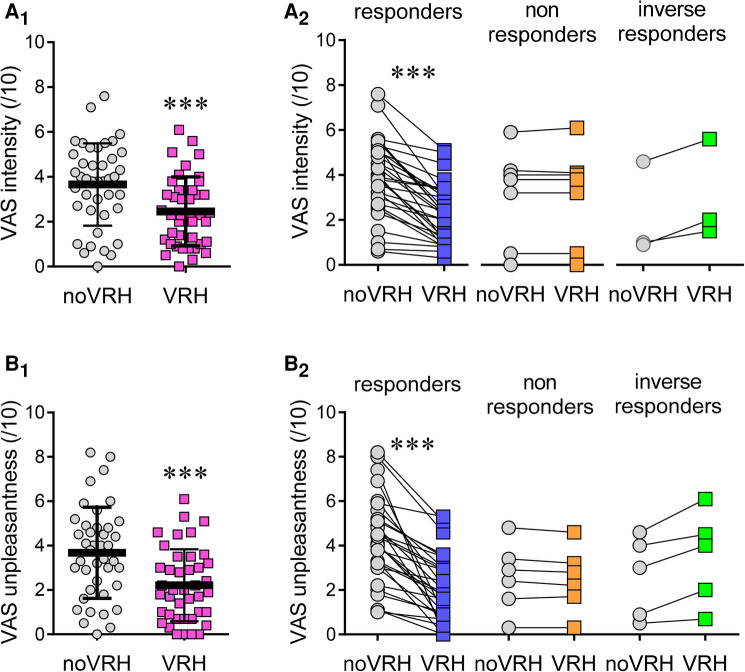
VAS scores (/10). (**A**) Mean VAS scores for pain intensity (**A**) and pain unpleasantness (**B**) when subject were submitted to cold stimulation at 4/10 with the VAS. Panels on the right (**A2**, **B2**) illustrates VAS changes for responders (10% decrease), non-responders (changes below 10%) and inverse responders (changes > 10%). Data selected for this graph were compared using the Student's *t*-test. Statistical code: (***), *p* < 0.001.

VAS scores were collected at the end of the calibration period, during each baseline preceding the noVRH and the VRH sequences (see design in Figure 1 and VRH comparisons in Figure 2). Mean intensity perception and unpleasantness between the different sequences were highly significant in the global statistics (one way ANOVA for intensity: F_(2, 78)_ = 19,38, *p* < 0.0001; and unpleasantness: F_(2, 77)_ = 23,40, *p* < 0.0001). They were statistically differences between the noVRH and VRH sequences for intensity perception (noVRH: 3.66 ± 1.83; VRH: 2.45 ± 1.53; Tukey posthoc test: *p* < 0.0001) and unpleasantness (noVRH: 3.67 ± 2.05; VRH: 2.21 ± 1.62; Tukey posthoc test: *p* < 0.0001). In good agreement, VAS scores were also significantly different between VRH and the calibration period Tukey's posthoc; Intensity: *p* = 0.0004; Unpleasantness: *p* < 0.0001) whereas no differences were found between the calibration period and the noVRH sequence (Tukey's posthoc; Intensity: *p* = 0.168; Unpleasantness: *p* = 0.594). In comparison with noVRH, VRH mean reduction of VAS intensity and unpleasantness score was of −1.20 ± 1.34 (range from −4.3 to +1.1) and of −1.46 ± 1.83 (range from −7.4 to +1.5), respectively. This revealed a high heterogeneity between subjects.

In more details, a sex specific difference in VAS scores was maintained after noVRH, for pain intensity (male: 2.95 ± 1.79, *n* = 22; female: 4.48 ± 1.56, *n* = 19; Student's *t*-test, *t* = 2.893, *df* = 39, 95% CI: 0,4599 to 2,598; *p* = 0.0062) and unpleasantness (male: 2.88 ± 2.01, *n* = 22; female: 4.60 ± 1.72; *n* = 19; Student's *t*-test, *t* = 2.910, *df* = 39; 95% CI: 0,5240 to 2,912; *p* = 0.0059). This was not the case after VRH (although a tendency can be seen) for pain intensity (Male: 2.032 ± 1.52, *n* = 22; Female: 2.95 ± 1.44, *n* = 19; Student's *t*-test, *t* = 1.981, *df* = 39, 95% CI: −0,01931 to 1,861; *p* = 0.0547) and unpleasantness (Male: 1.88 ± 1.7, *n* = 22; Female: 2.61 ± 1.48, *n* = 19; Student's *t*-test, *t* = 1.448, *df* = 39; 95% CI: −0,2891 to 1,745, *p* = 0.156). Overall, the VRH inhibitory effect was similar in men (*n* = 22) and women (*n* = 19) for the intensity (Male: −0.95 ± 1.89; Female: −1.65 ± 1.47; Student's *t*-test, *t* = 1.589, *df* = 18: 95% CI: −1,446 to 0,2300, *p* = 0.1295) and for pain unpleasantness (Male: −1.00 ± 1.87; Female: −1.93 ± 1.68; Student's *t*-test, *t* = 1,772; *df* = 39; 95% CI: −2,120 to 0,1401; *p* = 0.084).

There was a significant correlation between the hypnotizability score and the VRH change in pain intensity perception (Spearman *r* = −0.44; 95CI: −0.67 to −0.14; *p* = 0.0046; *n* = 41) but not for the change in unpleasantness. Apart from one person who did not like virtual reality because he felt claustrophobic wearing the headset, the remaining 40 subjects reported a positive experience with the VR disposal and script.

In order to analyze finely pain perception data, we arbitrary set a threshold at 10% change to identify possible subgroup of subjects and possible VRH non-responders. Out of 41 subjects, 31 (76%) reported a decrease in pain intensity perception under VRH by more than 10%, 7 subjects (17%) had pain intensity score changes below 10% and 3 subjects (7%) expressed an increase in pain perception above 10% with VRH (Figure 2A). This yields three subgroups, “Responders”, “Non responders” and “Inverse responders” respectively (Figure 2). While performing a similar selection using VAS score for unpleasantness, 73% of the subjects were from the responder group (15% non-responders, 13% inverse responders). As too few subjects were in the “Non-responders” or “Inverse responders” categories to allow statistical analyses, they were grouped (and referred from now on to as “non-responders”) in order to compare them with the responder's category for the remaining part of the article.

### VRH influences on physiological parameters

The analysis of cold pain-associated changes in cardiac parameters could only be done on 31 subjects. The first 8 subjects were drawn back from analysis because of hardware issues corrupting the simultaneous acquisition of physiological and VR signals. Two additional subjects were removed from the study because of excessive (not exploitable) electrical artifacts on the physiological recordings. We performed a repeated measure two-way ANOVA followed by a Sidak posthoc test (see values in Table 2 only for baseline and stimulation 4/10 sequences). On these subjects, we found no differences between VRH subgroups (all subjects, responders and non-responders) for mean heart rate, and pNN50 during baseline and stimulation periods (Table 2). Mean heart rate and pNN50 were significantly affected during the stimulation period but these changes were not specific of VRH. This was not the case for RMSSD whose mean value was significantly increased under VRH (compared to noVRH) during the baseline period and further more during the stimulation period under VRH [repeated measure two-way ANOVA, time x noVRH-VRH sequence, F_(1,29)_ = 16.76, *p* = 0.0003]. Interestingly, VRH-associated increase in RMSSD during baseline was found only among responders (Table 2).

**Table 2:** Changes in the mean values describing cardiac parameters (± SD) for heart rate, pNN50, RMSSD and ratio between low and high frequency during baseline (i.e. before stimulation) and during painful heat stimulation (4/10).

		noVRH		VRH	
		Baseline	4/10	Baseline	4/10
HR (bpm)	Mean (± SD)	63.9 (9.1)	61.3 (10.6)*	62.6 (8.9)°	59.9 (8.2)*
	Responders	62.8 (8.5)	60.7 (10.7)*	61.6 (9.3)	59.2 (8.3)*
	Non-Responders	67.7 (10.5)	63.3 (10.6)*	65.6 (7.7)	62.1 (8.1)*
pNN50 (%)	Mean (± SD)	17.7 (20.4)	19.5 (22.9)	20.9 (18.5)°	18.1 (18.9)
	Responders	20.4 (21.8)	22.8 (24.5)	23.6 (19.1)	21.2 (30.1)
	Non-Responders	9.3 (17.8)	9.0 (13.7)	12.5 (14.7)	8.3 (12.2)
RMSSD (ms)	Mean (± SD)	39.5 (26.2)	38.9 (26.4)	49.4 (26.8)°	39.4 (19.9)*
	Responders	42.4 (26.5)	42.0 (27.3)	53.2 (27.4)°	42.5 (19.0)*
	Non-Responders	30.2 (28.6)	29.6 (22.4)	37.0 (27.1)	29.1 (20.8)
LF/HF ratio	Mean (± SD)	2.0 (1.9)	1.9 (1.7)	2.9 (2.4)°	2.4 (2.2)°
	Responders	1.6 (1.7)	1.6 (1.6)	3.0 (2.4)°	2.5 (2.4)°
	Non-Responders	3.5 (2.2)	2.8 (1.9)	2.6 (1.4)	2.3 (1.3)

Significant differences with the Sidak's posthoc test at *p* < 0.05 are indicated as follow: °noVRH vs. VRH; *baseline vs. 4/10. VRH, virtual reality hypnosis; HR, heart rate; pNN50, percentage of successive NN separated by >50 ms; RMSSD, root mean square of successive differences.

Normalized low frequency (LF), normalized high frequency (HF) composing heart rate signal were next extracted and their mean ratio (LF/HF) shown in Table 2. Analysis yielded a higher LF/HF ratio observed under VRH during baseline and the stimulation period, compared to noVRH, for all subject and responders. No differences are seen between baseline and stimulation periods for non-responders.

### Respiration rate

**Figure 3:**
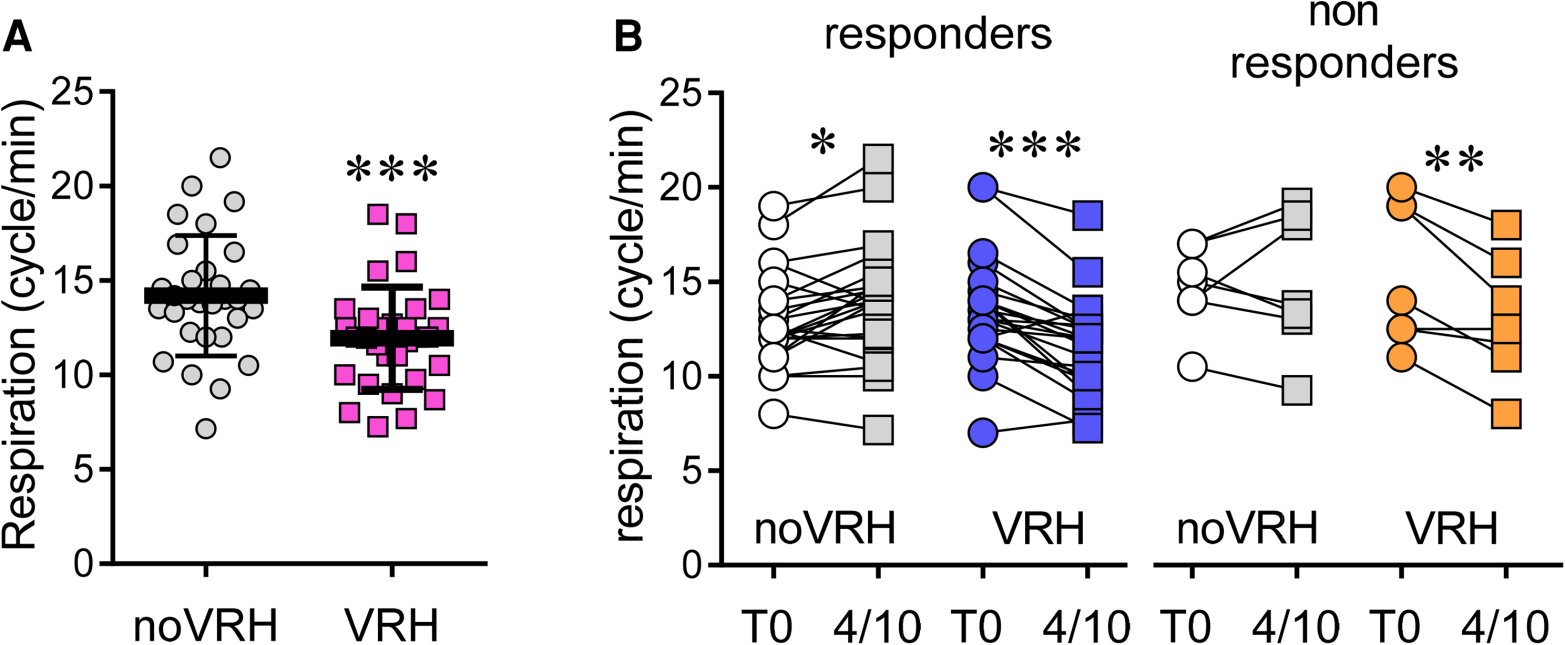
Respiration rate (cycle/min) at rest among the whole population (**A**) and, for to and VRH-noVRH, after segregation into responders and non-responders subgroups (**B**) statistical code for the paired Student's *t*-test: ***, *p* < 0.001; **, *p* < 0.01; *, *p* < 0.05.

As previously seen in other studies, VRH deeply affects respiration rates, which are significantly reduced and statistically different between groups (Figure 3A; all subject analysis). VRH condition is associated with reduced mean respiratory rate (mean reduction of −3.83 ± 2.65 cycles/minute; 95% CI: 1.82 to 5.85; Student's *t*-test: *p* < 0.0001; *n* = 29). Once again, this VRH induced reduction in the mean respiratory rate could be seen in the responder group but, surprisingly, also in the non-responder group (Figure 3).

Although responders had a slight yet significant increase in their respiration rate in noVRH condition (+0.92 ± 1.69 cycles/minute; 95% CI: 0,170 to 1,666; Student's *t*-test *p* = 0.019; *n* = 22), they had reduced respiratory rates in VRH condition (−1.99 ± 1.646 cycles/minute; 95% CI: −2,721 to −1,261; Student's *t*-test *p* < 0.0001; *n* = 22). Among non-responders, respiration rates remained stable in noVRH condition (+0.29 ± 2.28 cycles/minute; 95% CI: −1,818 to 2,395; Student's *t*-test: *p* = 0.75; *n* = 7), mean values were decreased in VRH (−2.39 ± 1.66 cycles/minute; 95% CI: −3,926 to −0,8600; Student's *t*-test: *p* = 0.0088; *n* = 7).

## Discussion

Our study shows that the VRH device tested here reduced perceived pain intensity and unpleasantness by 33% and 40%, respectively, on average, but not all subjects had a reduction in perceived pain with VRH. As an attempt to explain these different responses, we divided our cohort based on the percentage change in perceived intensity between noVRH and the VRH condition. This yielded three groups: responders (i.e., VRH reduced by more than 10% VAS pain scores: 75.6% of the subjects), non-responders (i.e., VAS pain score change between −10% and +10%), and inverse-responders (i.e., VAS pain score higher than +10%). However, because there were too few subjects in the non-responder and inverse-responder groups, they were grouped for the analyses and referred to as “non-responders”. Hypnotizability was found to be the most important predictor of VRH efficacy (i.e., in the responder group), as hypnotizability correlated with a reduction change in pain intensity below 10% from noVRH to VRH conditions. As expected, there was a sex-specific difference in hypnotizability scores as seen in previous studies (32). While women had higher scores than males during baseline and after VRH, subject sex/gender could not be predictive of VRH responsiveness in our study.

Interestingly, there were also significant differences in heart rate variability parameters between the responder and non-responder group. We found that RMSSD was the best predictor of VRH-induced analgesia. RMSSD was significantly higher in responders throughout the experiment, regardless of condition (noVRH or VRH). Breathing exercises administered by VRH during the baseline period resulted in an increase in RMSSD at that time point only in responders. Surprisingly, a decrease in HF (i.e., mostly reflecting parasympathetic tone) was observed in the VRH condition compared with the noVRH condition. This was associated with a higher LF/HF ratio in the VRH condition in responders, whereas this ratio did not change in non-responders. These data seem to indicate an increase in parasympathetic tone and thus a decrease in arousal under VRH compared with noVRH. One likely explanation is that VRH reduces the emotional processing by central nervous system structures leading to a predominance of the parasympathetic tone in the autonomic nervous system. Another synergistic possibility would be that VRH-induced reduction in respiratory rate contributes to reach a level of cardiac coherence. It is worth mentioning here that slow breathing was not always correlated with reduced pain perception (i.e., in the non-responder group). It is then unlikely that slow breathing could account for a major VRH-related analgesic effect. It remains, however, that slow breathing was only observed under VRH confirming the efficacy of the script and possibly contribution to the well-being of the subject.

As mentioned in the introduction, VRH research studies are still limited in number, and it is complicated to relate this work to other studies of VRH. Like Patterson and coworkers, we conducted this experiment with healthy volunteers to have better control over our population (in terms of age, sex, absence of disease, hypnotic ability, etc.) (14). They compared VRH with hypnosis and VR alone. Although they found a more positive effect of VRH than VR or hypnosis alone on induced heat pain in weakly hypnotizable subjects, there was no advantage of VRH (compared to hypnosis) in strongly hypnotizable subjects. These results may be explained by the induction method. Indeed, the induction in VRH was not performed simultaneously with the VR movie. However, it would have been of interest to test whether VR is important to induce hypnosis in low hypnotizable subjects. The work of Rousseaux and colleagues was performed with patients undergoing cardiac surgery (25). In contrast to our study and that of Patterson and colleagues, they found no effect of VR, VRH, or hypnosis on postoperative pain and anxiety. However, they did not conduct the experimental session during surgery, but one session the day before and one on the first postoperative day. The subjects were mainly men, and male gender may have a negative effect on hypnotizability, as mentioned earlier. In addition, the subjects were older, which has no effect on hypnotizability (33, 34), but it has been shown to decrease the effect of distraction on pain modulation (35).

This study has several limitations: First, our sample is highly educated (average 4.8 years after high school graduation), which has been shown to correlate with the efficacy of VR. Individuals with low levels of education are more likely to benefit from VR (36). Furthermore, education has a positive correlation on hypnotizability (37). This should be considered in future studies. In addition, we deliberately chose to conduct this exploratory study without a parallel “control” conditions, as it is difficult to imagine which control would be the most appropriate (2D VR, head-mount device with fixed images or luminosity, etc.). We made the choice of testing the VRH script as a proposal to reduce experimental cold pain in comparison to noVRH.

Altogether, and despite these limitations, this study is supporting the idea that some cardiac parameter may be predictive of a successful VRH-induced analgesia, when healthy subjects are submitted to cold pain. It also gives for the first time an idea of how much healthy subjects respond to VRH-induced analgesia while facing cold experimental pain. This temperature modality has not been tested so far with VR devices and this is promising for a possible clinical study with neuropathic patients who often exhibit mechanical and cold allodynia among other sensory symptoms.

## Data Availability

The original contributions presented in the study are included in the article/Supplementary Material, further inquiries can be directed to the corresponding author.
